# Dysbiosis in Pediatrics Is Associated with Respiratory Infections: Is There a Place for Bacterial-Derived Products?

**DOI:** 10.3390/microorganisms9020448

**Published:** 2021-02-22

**Authors:** Stefania Ballarini, Giovanni A. Rossi, Nicola Principi, Susanna Esposito

**Affiliations:** 1 Department of Experimental Medicine, University of Perugia, Didactic Pole “Sant’Andrea delle Fratte”, 06132 Perugia, Italy; 2 Unit of Pediatric Pulmonary, G. Gaslini University Hospital, 16147 Genoa, Italy; giovannirossi@gaslini.org; 3 Università degli Studi di Milano, 20122 Milan, Italy; nicola.principi@unimi.it; 4 Department of Medicine and Surgery, Pediatric Clinic, University of Parma, 43126 Parma, Italy; susannamariaroberta.esposito@unipr.it

**Keywords:** RTIs, wheezing, asthma, dysbiosis, microbiota, microbiome, children

## Abstract

Respiratory tract infections (RTIs) are common in childhood because of the physiologic immaturity of the immune system, a microbial community under development in addition to other genetic, physiological, environmental and social factors. RTIs tend to recur and severe lower viral RTIs in early childhood are not uncommon and are associated with increased risk of respiratory disorders later in life, including recurrent wheezing and asthma. Therefore, a better understanding of the main players and mechanisms involved in respiratory morbidity is necessary for a prompt and improved care as well as for primary prevention. The inter-talks between human immune components and microbiota as well as their main functions have been recently unraveled; nevertheless, more is still to be discovered or understood in the above medical conditions. The aim of this review paper is to provide the most up-to-date overview on dysbiosis in pre-school children and its association with RTIs and their complications. The potential role of non-harmful bacterial-derived products, according to the old hygiene hypothesis and the most recent trained-innate immunity concept, will be discussed together with the need of proof-of-concept studies and larger clinical trials with immunological and microbiological endpoints.

## 1. Introduction

Pediatric respiratory infections (RTIs) are a burden, as they represent up to 40% of the primary health care solicitations [1]. Upper respiratory tract infections (URTIs), including rhinitis, nasopharyngitis, tonsillitis, and laryngitis in addition to otitis media, comprise 88% of total respiratory infection episodes [2,3]. Lower respiratory tract infections (LRTIs), although occurring overall much less frequently than URTIs, are among the most common infectious causes of mortality worldwide. According to the World Health Organization (WHO), RTIs represent the fifth cause of death in children in low-mid income countries and the eighth in Europe [4]. RTIs are mainly of viral etiology with rhinovirus (RV), respiratory syncytial virus (RSV) and influenza virus (IV) being the most identified agents. Bacteria, such as *S. pneumoniae* and *H. influenzae*, frequently play a role in superinfections, which are often preceded by viral infection of the upper respiratory tract. Bacterial infection has been observed in 60% of patients with prolonged symptoms (≥10 days) of an URTI [5,6]. RTIs impose a heavy burden on the health care system, families and indirectly on the economy [7]. The frequent inappropriate antibiotic prescription for RTIs contributes to the raise of antibiotic resistance. Moreover, antibiotic treatment is known to disrupt the dynamic balance between the intestinal and upper airway microflora and host human immune cells, favoring acute or chronic illness [5,8].

Children are prone to RTIs, their recurrences and complications, mainly because of the immaturity of their immune system, physiological and genetic factors, as well as social and environmental factors such as family size, pollution, parental smoking and day care [9,10]. In developed countries, around 25% of children of less than 1 year of age, and ~18% of those aged 1–4 years, suffer from recurrent RTIs (rRTIs) [11]. In infancy and early childhood, severe viral RTIs most frequently caused by RSV or RV-A and C are associated with risk of recurrent wheezing and asthma [12]. Asthma is associated with morbidity and mortality, and its complex etiopathogenesis as well as phenotype heterogeneity, make its management challenging, despite the availability of effective maintenance treatments and guidelines. The identification of the main risk factors, as well as of patients at increased risk for this chronic inflammatory lung disease, is necessary for effective prevention and prompt care. In this context, recent findings pointed out the role of the complex interplay between host microbiota and the immune system in determining chronic respiratory pediatric conditions. Human microbiota influences both the development of mucosal and/or epithelial barriers and modulates the immune response, thus contributing to immune homeostasis. Despite the increasing evidence of the association between the commensal communities’ dysregulation with a number of immune-mediated diseases, including respiratory infective and allergic conditions, the specific underlying mechanisms and inter-talks between human immune cells and microorganisms, the “meta-organism”, as well as their metabolites, still need to be fully elucidated [13]. Understanding the processes involved in bacterial gut/airways changes and immune vulnerability might allow the identification of potential biomarkers in at-risk patients. This might promote the study of alternative interventions and precise medicine strategies, aimed at reducing the risk for recurrent and chronic infectious respiratory conditions [14,15]. Modulating the immune system activity in a non-specific manner in pediatric patients has been suggested as a complementary strategy to improve protection against rRTIs and their consequences. Referring to the hygiene hypothesis and the recent discoveries on “farm-dust” effects [16], some authors have suggested that microbial-derived products can have a “farming” effect on microbiota and promote an efficient immune response [17,18,19].

A literature search was performed in PubMed using pre-defined keywords (e.g., microbiome, microbiota, respiratory disease, airways, infection, bacteria, bacterial species, atopy, allergy, wheezing, asthma, children) and limiting the hits to the past 10 years. Only articles in the English language or with an abstract in English and from peer-reviewed journals were retained.

Our review summarizes the most recent scientific knowledge on gut and airways dysbiosis in pediatric respiratory infections and asthma. In detail, it addresses the main interrelation between microbiota and immune response at mucosal level as well as the associations between gut/airways bacterial community disruption and respiratory infectious diseases. It ultimately discusses the place of bacterial-derived products in pediatric dysbiosis and associated respiratory disorders.

## 2. Gut and Airway Microbiota and Immune Homeostasis: Key Players, Interactions and Mechanisms Overview

**Figure 1:**
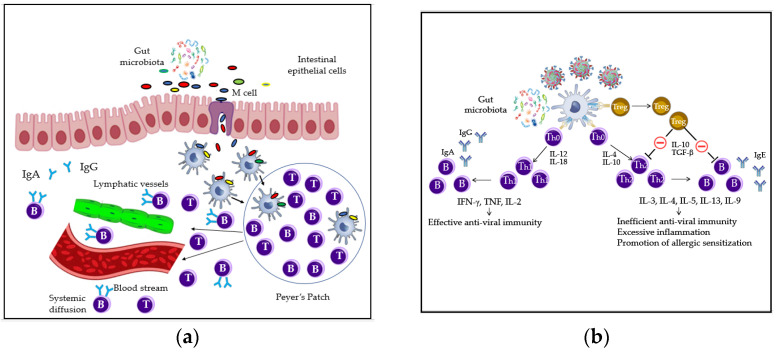
Recognition of commensal and IgA production and Treg cell-mediated suppression of an inefficient adaptive Th2-type immune response: (**a**) gut microbiota antigens are taken up by Microfold cells (M) cells and then processed by DCs (dendritic cells) in the gut-associated lymphoid tissue. DCs stimulate the local immune response in the PPs (Peyer’s Patches) with subsequent migration of immune effector cells via blood and lymphatic vessels to other organs [21]; (**b**) in viral infections, through the release of IL-10 and TGF-β, Treg cells inhibit Th2 lymphocyte maturation from Th0 lymphocytes and the release of Th2 cytokines, thus repressing IgE production by B-cells. Moreover, Treg cells are also involved in the promotion of viral clearance but also in excessive Th1-type polarization of the immune response [23]. These lead to an effective anti-viral immunity and prevention of allergic sensitization [24].

Humans are colonized by trillions of commensal bacteria known as microbiota, characterized by high interpersonal variation in the first year of life and influenced by several factors, such as mode of delivery, exposure to pathogens and antibiotic use [20]. Immune homeostasis has been extensively studied at the intestinal level in humans; it is well-accepted that the complex interrelations between structural and immune cells and commensal microorganisms, as well as pathogens, are responsible for the immune system’s healthy status. Indeed, intestinal epithelial cells offer a physical barrier towards microbes, produce specific antimicrobial peptides and are engaged in several cross-talks with microorganisms and/or immune cells for example dendritic cells (DCs). Together with specific immune players, IECs act as sentinels by doing a triage amid commensals and pathogens through recognition of microorganism- or pathogen-associated molecular pathways (MAMPs and PAMPs), as well as by presenting the pathogens to other antigen presenting cells (APCs), i.e., macrophages and DCs. The selective non self-recognition process in the epithelial layer or in the underneath tissue is promoted by the basolateral or intracellular (endosome) localization of some pattern recognition receptors (PRRs), with Toll-like receptor 4 (TLR4) and Nucleotide-binding oligomerization domain-containing protein 1 and 2 (NOD1/NOD2) being the most common. While the expression of receptors such as TLR2 and TLR4 is limited in steady-state conditions, it is increased in the presence of “dangerous” signals. Conversely, the recognition of commensals and their metabolites is known to activate an intracellular cascade at the gut mucosa level, a highly specialized barrier where immune and structural cells act synergistically to maintain the immune homeostasis. In detail, self-recognition induces the inhibition of the NF-kB translocation into the nucleus, so the transcription and release of cytokines leading to a pro-inflammatory state. Gut microbiota can stimulate DCs in the Peyer’s Patches (PPs) to directly activate B cells, leading to antibodies IgA production through class switching; this process reduces the risk of food allergy (FA) that is caused by low IgA levels at the intestinal surface barrier, as shown in Figure 1a. Low microbiota abundance appears indeed to be related to low immunoglobulins A (IgA) production [21]. Furthermore, self-recognition of commensal bacteria leads to expansion of T cells with regulatory capacities (Tregs), a subpopulation of T cells that modulate the immune system, maintain tolerance to self-antigens and prevent autoimmune diseases (Figure 1b) [22]. In viral infections, Treg cells are involved in the process of viral clearance as well as modulation of many aspects of the innate and adaptive immune response. They contribute to the prevention of an excessive antigen-specific CD4+ and CD8+ T-cell response and, importantly, to the limitation of inappropriate and inefficient Th2- type immune reaction, through the production of the inhibitory cytokines IL-10 and TGF-β [23]. Overall, the gut microbiota is deeply involved in the homeostatic balance between tolerance and immunity. It plays unique regulatory functions on the mucosal immune system, inhibiting or activating specific intra-cellular molecular pathways aimed at dampening overt inflammatory responses against the gut microbiota [24].

**Figure 2:**
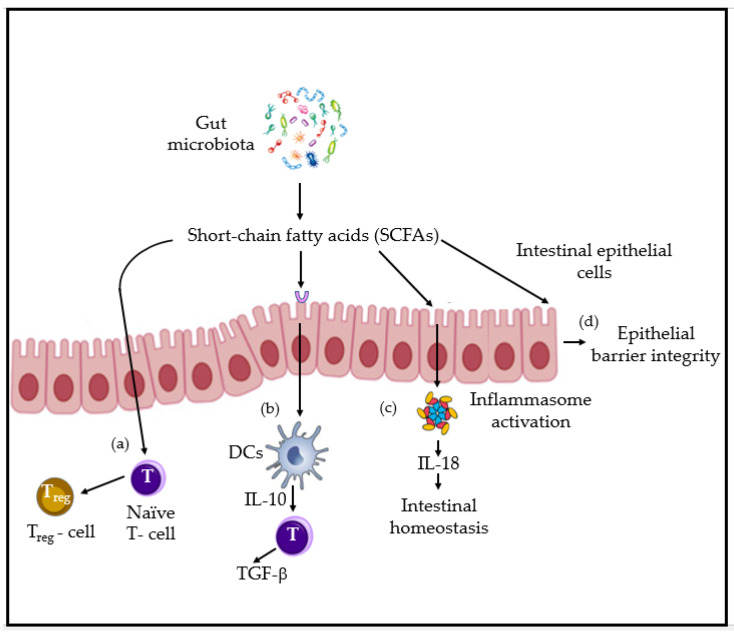
Short-chain fatty acids (SCFAs) acetate, butyrate and propionate produced by gut microbiota promote: (**a**) the differentiation of naïve T cells into Treg cells; (**b**) the activation of DCs to induce TGF-β release by activated T cells; (**c**) intestinal homeostasis, through inflammasome activation (release of IL-18); (**d**) the production of intestinal antimicrobial peptides (AMPs) and epithelial barrier integrity [13,25].

Several reciprocal feedback loops between the most studied gut microbiota and metabolites and the innate immune system receptors and effectors have been recently described [25]. Amongst short-chain fatty acids (SCFAs), long-chain fatty acids (LCFAs), polysaccharides, amino acids and vitamins; the former ones are considered the most relevant. Human beings cannot digest on their own some polysaccharides, such as cellulose from edible plants. SCFAs of 1–6 carbon length are the main metabolites of commensal gut bacteria anaerobic fermentation of indigestible polysaccharides such as dietary fiber and resistant starch. The most prevalent and studied for their roles are acetate (C2), propionate (C3) and butyrate (C4). Acetate is produced, for example, by *Akkermansia muciniphila*, *Bacteroides* spp., *Bifidobacterium* spp., *Prevotella* spp., *Ruminococcus* spp., *Clostridium* spp., *Streptococcus* spp. Propionate is synthetized by *Bacteroidetes* spp., *Roseburia* spp., *Firmicutes*, *Ruminococus* spp., *Clostridium* spp., *Eubacterium* spp., *Coprococcus* spp., and *Akkermansia muciniphila*. Butyrate biosynthesis is done by *Roseburia intestinalis* and *insulinivorans*, *Eubacterium rectale*, *Clostridiales bacterium*, *Coprococcus* spp., *Clostridium symbiosum*, *Faecalibacterium prasnitzii* and *Bacteroidetes* spp. SCFA biosynthesis pathways, as well as the main receptors and tissue localization, were reviewed by Ratajczak et al. [13]. These acids are known to maintain proper microbiota dynamics through inhibiting proliferation of some bacteria at a low pH environment [26]. In addition, SCFAs regulate several immune functions via molecular mechanisms that involve both epithelial and immune competent cells. They affect immune reactions not only in the intestines, but also in distant tissues. They have been described to enhance chemotaxis of neutrophils and increase cytolytic activity [25], reduce pro-inflammatory cytokine production of macrophages by inhibition of histone deacetylases, and epigenetically remodel the FoxP3 locus in regulatory T cells to promote Treg cells expansion [13]. In Figure 2, the effects of SCFAs are described.

**Figure 3:**
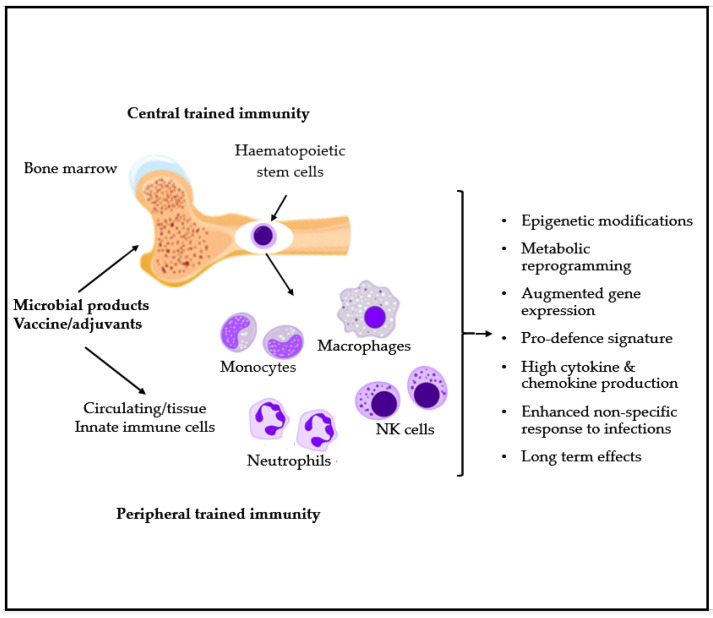
Microbial-derived products as well as some inactivated or attenuated microbial products are suggested to promote the innate immunity training both at peripheral and central level in the bone marrow on HSC (hematopoietic stem cells). This involves epigenetic modifications, modified gene expression, metabolic reprogramming and a pro-defense signature characterized by increased chemokines and cytokines production [32].

*Clostridium* spp., *Streptococcus* spp. *Bacteroides* spp. and *Bifidobacterium* spp. were described as able to produce acetate [13,27], while the major producers of butyrate are found among the *Firmicutes* (*Lachnospiraceae* and *Ruminococacceae*) with some species in the *Lachnospiraceae* family such as *Roseburia inulinivorans* able to also produce propionate. The major producers of propionate are found in the phyla of *Bacteroidetes* (*Bacteroidaceae* and *Prevotellaceae*) [28]. The integrity of the gut mucosal barrier and immune function, as well as of its commensal community, is a key factor to maintain a general healthy status, as any alteration of microbiota and its metabolic products in the intestine affects other mucosal activities. Associations have been found between the composition of the gut and lung microbiota, known to function as a single “organ”, and the risk of to develop respiratory and/or allergic diseases [21,29,30]. The nature of bacterial composition in the airways can affect the immune homeostasis and the inflammatory responses to infections. This can be determined by direct recognition of bacteria by the immune cells, as well as by bacterial metabolites released in the intestine that reach via bloodstream the airway structures influencing the local immune response [31]. For example, gut SCFAs are known to have a favorable effect in the airway mucosa protecting from exacerbations in allergy [32]. It is known that exposure to selected vaccines, such as bacillus Calmette–Guerin (BCG) or microbial components, can increase the baseline tone of innate immunity and trigger pathogen-agnostic antimicrobial defense. De Laval et al. showed also a long-term innate immune memory that would be driven by myeloid cells (monocytes, macrophages and neutrophils) [33]. Trained innate immunity might lead to a faster and increased responsiveness upon contact with a pathogen, thus decreasing the severity of the infection and potentially limiting its spreading (Figure 3) [34].

Clarke et al. [35] observed that gut microbiota primes systemic innate immunity thanks to the translocation of the microbiota diaminopimelic acid-type peptidoglycan (DAP-PG) from the intestine to the circulation. DAP-PG showed to prime neutrophils via NOD1 receptors in the bone marrow (epigenetic effect), increasing their capacity to kill microorganism, regardless of their location in the host mucosa.

**Figure 4:**
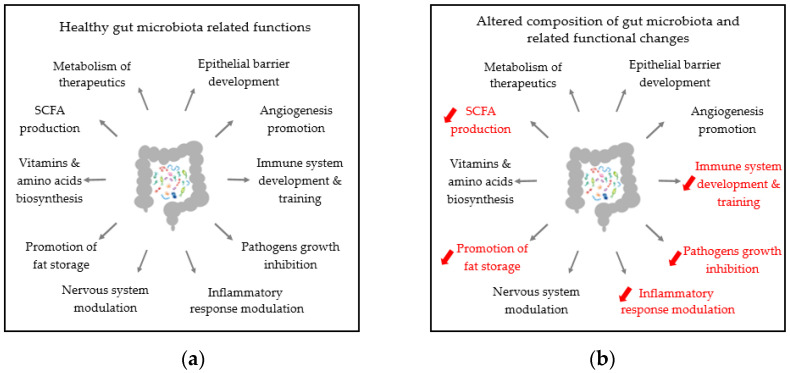
Microbiota functions affect different host systems [36]: (**a**) list of benefits derived by healthy gut microbiota functions; (**b**) examples of main negative consequences due to the altered composition of the gut microbiota partially due to lifestyle and habits factors.

Gut microbiota provides several services as described in Figure 4a, but it is highly vulnerable. Along with the shift in microbiota composition caused by industrialization and associated lifestyle habits, certain activities have been lost or become inefficient, leading to diseases as shown in Figure 4b [36]. Gut commensal community and immune homeostasis impairment have been associated with several chronic inflammatory states affecting different systems.

As well as the gut, the airways harbor bacterial communities whose composition, richness and relative abundance can diverge from what is considered a “healthy status.” These modifications can be associated or responsible for a number of immune diseases including respiratory infective, allergic conditions and chronic inflammatory lung diseases. If, on the one hand, the respiratory microbiota composition early in life influences the predisposition to respiratory disorders and eventually their recurrence and severity, on the other hand, a heathy airways microbiome can be impaired by the number of prior experienced respiratory infections [29]. The interactions between airway microbiota and immune response are similar to the ones already described for the gut [37]. In detail, a growing body of literature has demonstrated that the nasopharyngeal microbiota plays an important role in maturation and homeostasis of the host immune response in both the upper and lower airways. The balanced competitive/synergistic interplay between commensal and potentially pathogenic taxa, which is the hallmark of a healthy status, can be altered by the acquisition of new pathogenic virus and bacteria, increased virulence or prevalence of potential pathogens and/or decreased efficiency of host defenses, which can be the cause but also the effect of altered microbial communities [38,39,40,41].

## 3. Gut and Airway Microbiota Composition

**Figure 5:**
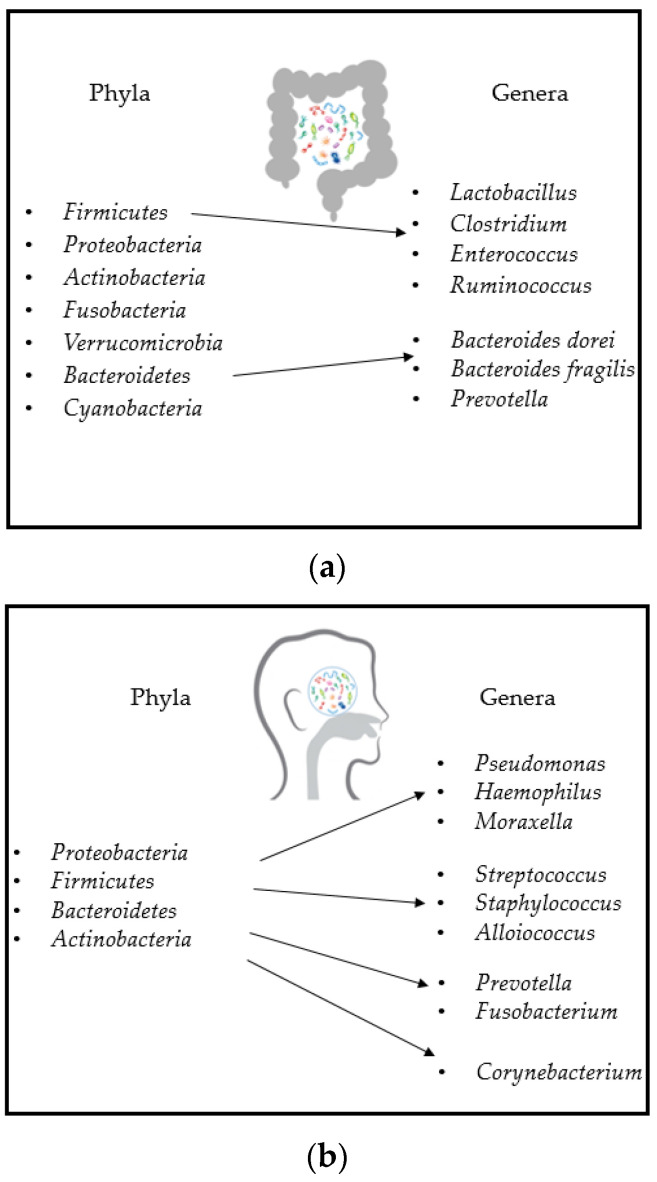
Microbiota composition: (**a**) main phyla and genera of a healthy gut microbiota [42]; (**b**) main phyla and genera of a healthy airway microbiota [44].

The number of microorganisms inhabiting the GI tract has been estimated to exceed 10^14^. Despite extensive sequencing efforts, the complete bacterial repertoire of the human gut microbiota remains undefined. In the gut, several phyla are present, such as *Actinobacteria*, *Bacteroidetes*, *Cyanobacteria*, *Firmicutes*, *Fusobacteria*, *Proteobacteria*, *Saccharibacteria*, *Spirochaetes, Synergistetes, Tenericutes* and *Verrucomicrobia*. The dominant ones are: *Firmicutes* (up to 70%), *Bacteroidetes* (up to 30%), *Proteobacteria* (<5%) *Actinobacteria* (<2%)*, Fusobacteria* and *Verrucomicrobia* (<1%), with *Firmicutes* and *Bacteroidetes* representing 90% of gut microbiota taxa. The *Firmicutes* phylum is composed of more than 200 different genera such as *Lactobacillus*, *Clostridium*, *Enterococcus* and *Ruminococcus*. *Clostridium* spp. represents 95% of the *Firmicutes* phylum. *Bacteroidetes* consists of predominant genera, such as *Bacteroides* (e.g., *Bacteroides dorei* and *fragilis*) and *Prevotella* (Figure 5a) [42].

Airway microbiota is an ecological niche that has only recently been studied. It is one order of magnitude lower than the intestinal one in healthy individuals, as it harbors a bacterial load in the order of 2000 genomes/cm^2^ of tissue, which is comparable to the upper two-thirds of the intestinal tract [43]. However, despite the lower bacterial load, the respiratory microbiota has a relative high taxa diversity. The initial source of the airway microbiota is thought to be the combination of environmental exposure and of interaction with the intestinal microbiota because dietary changes were described influencing its composition. In detail, it is possible that the bacteria found in the airways represent a transient community that is steadily repopulated via inhalation, gastric reflux or oropharyngeal micro-aspiration. Here, the most abundant phyla and genera are *Proteobacteria* (genus *Pseudomonas*, *Haemophilus, Moraxella*), *Firmicutes* (genus *Streptococcus*) and *Bacteroidetes* (genus *Prevotella* and *Fusobacterium*) [44]. At genus level, this was initially defined as the “core airway microbiota”, which should be similar in healthy people. Other genera are *Staphylococcus* and *Alloiococcus* (*Firmicutes*) and *Corynebacterium* (*Actinobacteria*), as shown in Figure 5b.

Several factors are known to influence the richness and the abundance of microbiota during the first years of life, such as prenatal maternal transfer, delivery modality, feeding/diet, intestinal environment (genetically or anatomically determined), age, immunity/microbial exposure/infections and antibiotic use [45].

## 4. Gut and Airways Dysbiosis in Respiratory Diseases

The imbalance of the host commensal microorganisms that leads to altered microbiome functions has been associated with compromised immune homeostasis and disease status or alteration of host physiology.

### 4.1. General Features of Gut and Airways Dysbiosis

Even if the mechanisms involved are still to be fully elucidated, it is known that gut dysbiosis may disrupt mucosal immunological tolerance. In healthy gut microbiota, bacteria belonging to *Firmicutes* are more enriched than *Bacteroidetes*, while the opposite was reported in gut dysbiosis according to population studies. The shift in the relative abundance of these two main phyla causes a reduction of SCFA production and an increase in some inflammatory cytokines, such as IL-6, IL-8 and TNF-α [46]. Several diseases have been associated with gut dysbiosis-dependent alteration of the immune balance, such as Crohn’s disease, inflammatory bowel disease and syndrome, atherosclerosis, cardiovascular disease, multiple sclerosis, atopic dermatitis, psoriasis, chronic lung disease (i.e., asthma) with increased risk for airways infections and other metabolic disorders (pre-diabetic state, insulin resistance and obesity) [47].

**Figure 6:**
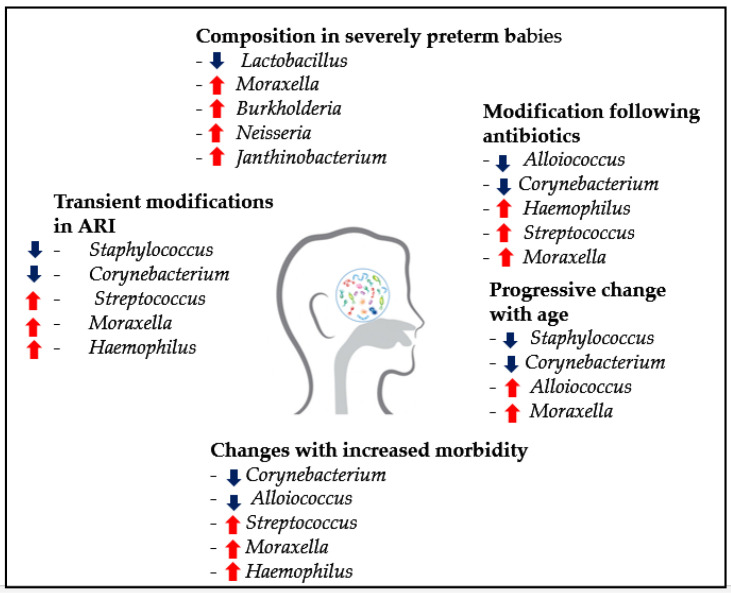
Non-exhaustive description of relative abundance of specific genera in the nasopharynx and changes associated with known influencing factors (e.g., age, antibiotic use and acute RTIs (respiratory tract infections)) [29]. Arrow up = associated with increase. Arrow down = associated with decrease.

In the airways, dysbiosis is mainly characterized by high prevalence of bacterial families such as *Moraxellaceae*, *Pasteurellaceae* and *Streptococcaceae* and the paucity of *Staphylococcaceae*. Several factors may influence the taxonomic bacterial composition in the airways. The most studied are: age, respiratory morbidity and use of antimicrobials. Figure 6 illustrates the main factors influencing the airway microbiota in children.

### 4.2. Dysbiosis and Respiratory Diseases

**Figure 7:**
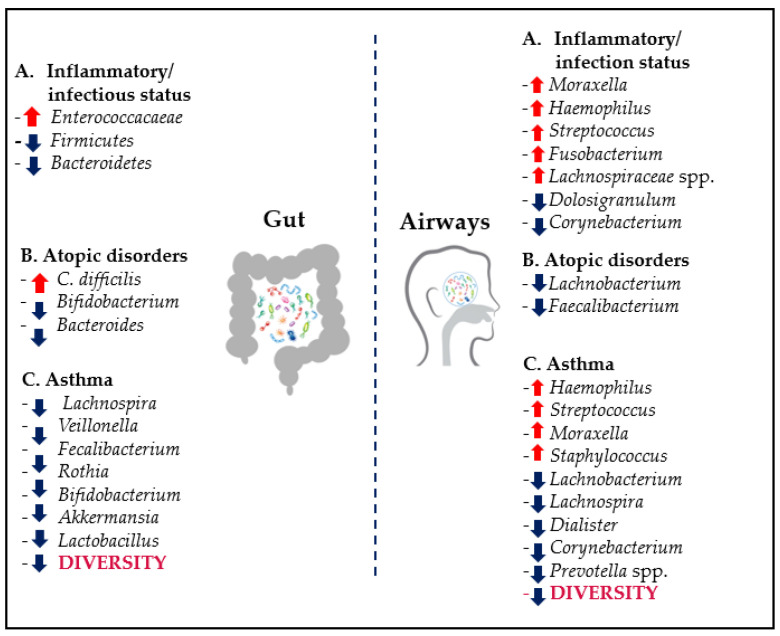
Overview of the main associations between changes in relative abundance of taxa in both gut and airways in children, and RTIs/inflammation, atopic disorders and asthma. Arrow up = increase. Arrow down = decrease

Dysbiosis of both gut and airways has been described in pediatric respiratory disease. In the present review, we address the association of microbiota profile changes in children and RTIs, their recurrences and chronic complications such as atopic respiratory disorders/asthma. An overview is shown in Figure 7.

#### 4.2.1. Pediatric RTIs and Recurrences

Although commensal bacteria are crucial in maintaining immune homeostasis of the intestine, their role in immune responses at other mucosal surfaces is accepted but remains less clear. A recent study examined the role of gut commensal bacteria in the initiation of adaptive immunity after respiratory infection with IV. It was observed that there is a need for an intact commensal gut bacterial community in the establishment of Th1, cytotoxic T-cell and IgA responses to the infection. Moreover, specific bacteria in the gut were fundamental for lung immunocompetence, such as the neomycin-sensitive gut bacteria. Their protective effect was partly mediated by the capacity to provide signals for robust priming of pro–IL-1β and pro–IL-18 expression at steady state in animal models [37]. In early life, gut dysbiosis is characterized by a decrease in individual diversity in the microbial ecosystem, with delays in the colonization of anaerobes and enhanced colonization by opportunistic pathogens, in particular *Enterobacteriaceae* and enterococci [48]. In a small study conducted by Li et al. in children with rRTIs, the investigators showed differences in six phyla (*Firmicutes*, *Proteobacteria*, *Bacteroidetes*, *Actinobacteria*, *Verrucomicrobia* and *Tenericutes*) and in four genera (*Enterococcus*, *Faecalibacterium*, *Bifidobacterium* and *Eubacterium*). In detail, *Enterococcus* was significantly more enriched in the rRTIs group, whereas the abundance of *Eubacterium*, *Faecalibacterium* and *Bifidobacterium* was significantly reduced in the rRTIs group [49].

Respiratory microbiome was described as having a protective role against RTIs of viral origin or, conversely, as favoring both infections and complications. The mechanisms are still object of intense study. *Dolosigranulum* and *Corynebacterium* dominant profile might be protective in early life; on the contrary, a high relative abundance of *Moraxella, Haemophilus* and *Streptococcus* has been associated with increased risk for RTIs and inflammatory sequelae. As shown by Teo et al., this profile might also be associated with more severe early RTIs, wheezing illness and asthma later in life [29]. In particular, Bosch et al. showed that children experiencing a higher number of RTIs in the first year of life had an aberrant microbial developmental trajectory starting from the first month of life compared with children with <2 RTIs per year. These patients showed a decreased microbial community stability, a prolonged reduction of *Corynebacterium* and *Dolosigranulum*, and the enrichment of *Moraxella* since early life species [50].

Similarly, Robinson et al. reported that the most common bacteria cultured in wheezing children were *Streptococcus pneumoniae*, *Moraxella catarrhalis*, *Staphylococcus aureus* and *Haemophilus influenza*. In addition, they showed that *Moraxella* species dysbiotic microbiota cluster was associated with airway neutrophilia, while a “mixed” microbiota cluster enriched in *Streptococcus*, *Prevotella*, *Neisseria* and *Porphyromonas* spp. was associated with a macrophage- and lymphocyte-predominant inflammatory profile [51]. In a report by Leung et al., the respiratory microbiome of IV infected and demographically comparable but not infected subjects showed differences that were associated with less mucociliary activity and lower secretion of immumodulatory metabolites. As a result, the host immune system response in the infected patients was described as lowered and leading to poorer clinical outcomes [52].

If, on the one hand, the respiratory microbiota composition early in life influences the progression of URTIs to the lower respiratory tract and the severity of the infection as well as the risk for future development of wheezing illness and/or asthma inception, on the other hand, the number of prior RTIs affects a healthy nasopharyngeal microbiome, thus leading to a self-perpetuating vicious cycle [29]. A virus-specific microbiota profile (or “signature”) has also been identified, showing distinct taxonomic patterns for the two most common respiratory viral infections caused by RSV and RV. It is hard to conclude whether viruses induce some changes in the nasopharyngeal microbiota or whether alterations already present before viral RTI can promote RSV or RV infections, although there is a strong rationale in favor of the first hypothesis. It is, indeed, well known that viruses might enhance the bacterial adhesion capacity and colonization even if some contradicting results came from other research [53,54]. Viral infections may promote high carriage in the URT of *Streptococcus pneumoniae*, whose presence has been associated with respiratory complications due to its possible migration to the middle ear or the LRT. Therefore, interaction between respiratory viruses (i.e., RSV, RV and IV) and respiratory microbiota at the time of the infection may modulate the immune response, thus potentially affecting the course of the disease (i.e., severity, recurrences and even bacterial complications) and consequently respiratory health in the long run [55]. The other way around, the predominance of some genera or species of the airway microbiota have been associated with protection, while others have been associated with higher morbidity or risk of respiratory complications as described by Pichon et al. [45]. Species associated with protection against severe viral RTIs were mainly *Staphylococcus aureus, Lactobacillus helveticus* and *rhamnosus*, *Veillonella*, *Escherichia coli* and *Prevotella* spp. while the ones that were associated with complications were *Haemophilus* spp. (mainly *Haemophilus influenzae*), *Streptococcus*, *Moraxella*, *Fusobacterium* and *Lachnospiraceae* spp.

*Haemophilus influenzae* was not only associated with complications but also with an increased risk for new viral infection. The mechanism behind this seems to be the increased expression of ICAM-1, a membrane receptor for RV, by bronchial epithelial cells, favoring viral binding and increased virulence. As already discussed by Teo et al. [29], the nasopharyngeal microbiome dominated by *Haemophilus influenzae* and *Streptococcus pneumoniae* in asymptomatic patients was also associated with an increased risk for RSV infection and with respiratory wheezing illness. The severity and the morbidity of such RTIs is mainly due to an exaggerated host inflammatory response (i.e., increased in airways and systemic pro-inflammatory cytokines). This was further observed in a cohort study of children less than two years of age, where the predominance in the respiratory microbiome of *Haemophilus* and *Streptococcus* have been associated with overexpression of TLR gene pathway as well as of genes implicated with neutrophil responses during RSV infections [56]. Concisely, the respiratory microbiome is responsible for controlling viral adherence on epithelial cells, or for activating via TLRs and DCs the innate immunity that limits the viral infection. At the same time, it might act on the innate immune system, promoting less destructive macrophage activities, and on adaptive immunity, promoting the production of IgA to control the inflammatory response. The respiratory microbiota of infants could be considered as a biomarker to identify the subjects at risk for more severe viral RTIs and wheezing at 5 years of age and to suggest appropriate interventions [57].

#### 4.2.2. Pediatric Asthma

Recent knowledge on the gut microbiome and its metabolic pathway has led to a better understanding of how important gut dysbiosis affects the immune system and contributes to pediatric asthma development. The reduction of SCFAs due to gut dysbiosis alters both the host immune response (skewing towards Th2 type inflammation) and the host energy metabolism (leading to rapid weight gain). When this happens early in life, gut dysbiosis predisposes to atopy-based and obesity-based asthma, respectively [58]. In addition, viral RTIs activate immune and structural cell in the airways promoting inflammation and influencing the responses to other pathogens, allergens and pollutants [59].

Moreover, following sensitization, allergen presentation by airway dendritic cells (DCs) induce a shift toward a Th2-type response. Therefore, when viruses infect epithelial cells and stimulate the release of Th2 pro-inflammatory chemokines, they further attract Th2 cells into the airway, which, in turn, secrete IL-4, IL-5 and IL-13. IL-5 promotes eosinophilia, and eosinophils themselves release other inflammatory mediators, such as transforming growth factor-β (TGFβ), inducing inflammation in the airway smooth muscle. IL-4 and IL-13 cause antibody class switching to IgE in B cells, which, after crosslinking with the allergen, bind mast cells and promote cell degranulation. In addition to histamine, prostaglandin (PGD2) and leukotrienes (LTC4, LTD4 and LTE4), mediators that cause bronchoconstriction and further airway inflammation, mast cells also produce the Th2 cytokines IL-4 and IL-13, further promoting Th2 type immune responses [60]. Galazzo et al. could identify microbial taxa that were differentially abundant among infants who did or did not develop allergic disease manifestations. *Lachnobacterium* and *Faecalibacterium* were significantly decreased throughout infancy among children who developed atopic dermatitis and among children at risk for allergic wheeze at the age of 1 year. The presence of *Clostridium difficile* was observed as associated with a significant increased risk for atopic dermatitis at least when colonization started by the age of 1 month [61,62]. Additionally, *Lachnospira* and *Dialister*, in addition to *Lachnobacterium*, were significantly decreased among children who developed asthma. The fact that these bacterial taxa were not only differentially abundant at a single time point but throughout infancy strengthens the likelihood of a causal role in the protection against allergic disease [63]. In line with these results, in the CHILD (Canadian Healthy Infant Longitudinal Development) study, the reduction of *Lachnospira*, *Veillonella*, *Faecalibacterium* and *Rothia* in the gut was detected in children who developed asthma at the age of 3 years [64]. This was confirmed by the WHEALS (Wayne County Health, Environment, Allergy and Asthma Longitudinal Study) study, which, in children who developed asthma at 4 years of age, showed a lower relative abundance in fecal sample at the age of 1 month of *Bifidobacterium* (*Bifidobacteriaceae*), *Lactobacillus* (*Lactobacillaceae*), *Faecalibacterium* (*Clostridiaceae*) and *Akkermansia* (*Verrucomicrobiaceae)*, as well as higher relative abundance of fungi such as *Candida* and *Rhodotorula*. Furthermore, a specific fecal metabolome enriched for pro-inflammatory metabolites, was associated with increased risk of subsequent asthma [65,66]. The presence of *Bifidobacterium* has been considered beneficial, while the specific colonization by *Bacteroides fragilis* at the age of 3 weeks has been suggested as an early indicator of possible asthma later in life [67]. Different results were reported in the literature, with authors showing that the administration of the purified capsular polysaccharide from this commensal bacterium *Bacteroides fragilis* could suppress the production of IL-17, stimulate CD4+ T lymphocytes to produce IL-10 and protect the colon mucosa against inflammatory reactions initiated by bacterial antigens [68]. By adjusting the balance between Th2 and Th17 patterns, the microbiome may play a role in controlling asthma endotype polarization in the airways, triggered by neonatal respiratory infections [69]. Indeed, absence of lung microbiota in germ-free mice was associated with an increase in Th2 response and allergy or asthma. Low microbial diversity in the lung with an increased proportion of *Proteobacteria* has been found in patients with asthma. In detail, colonization of the airways by some bacteria, such as *Haemophilus influenzae*, *Streptococcus pneumoniae*, *Staphylococcus aureus* and *Moraxella catarrhalis*, was associated with higher risk of asthma and its exacerbations, suggesting some synergistic mechanisms between pathogenic viruses and bacteria [70]. Hilty et al. conducted a systematic study on microbiota in the nasopharynx, oropharynx and lower airways of children with asthma. The asthmatic group was characterized by relative abundance of *Haemophilus* and *Staphylococcus* spp. (*Proteobacteria*) and reduced *Prevotella* spp. (*Bacteroidetes*) compared to controls [44]. In other studies in asthmatics, *Proteobacteria* were increased while *Firmicutes* and *Bacteroidetes* were reduced. In particular, *Prevotella* genus (*Bacteroidetes*) and *Corynebacterium* (*Actinobacteria*) were reduced in asthma [71]. Although there are several observations suggesting that specific taxa enrichment or reduction could be potentially associated with disease inception, it is still too early to fully define a healthy respiratory microbiota and to identify species that can help to preserve the homeostasis, as well as to confirm the causality and the mechanisms behind it [72].

## 5. Microbial-Derived Products as Possible Interventions in Pediatric Respiratory Recurrence and Asthma

Because gut dysbiosis and specific microbiota profiles association with asthma, any orally given product, such as prebiotics or probiotics, aimed at preventing or correcting microbiota impairment and the related dysregulation of important functions, could be considered a reasonable therapeutic approach. The oral administration of these products has been also thought to influence the airway microbiota in two ways: indirectly, by the release of bacterial metabolites that may promote the overgrowth of beneficial commensals; or directly thanks to a micro-aspiration of the probiotics from the digestive tract into the airways. The same mechanism could apply to other products given orally, such as bacterial lysates, which might mimic the exposure to bacteria in a natural way and so promote the immune homeostasis in the gut and subsequently in the lung [23].

In preclinical studies in a specific pathogenic-free mouse model, the colonization of URT by commensal bacteria species significantly attenuates influenza-mediated lung immune injury inducing the release of anti-inflammatory cytokines by M2 alveolar macrophages [73]. Moreover, in animal models, *Lactobacillus* demonstrated immunoregulatory effects in the lungs, confirmed by some promising evidence obtained in clinical trials by the administration of one specific strain: *Lactobacillus reuteri* [74]. Clinical studies have been performed to evaluate the effects of other probiotic treatments on allergic inflammation. Several different strains of *Lactobacillus* (*L. casei*, *L. acidophilus* strain L-92 and *L. paracasei* strain L-33) have been tested for their impact on symptoms severity during allergic rhinitis. All of these studies showed beneficial effects of the oral administration of live *Lactobacilli*-supplemented milk, such as a reduction in the number of rhinitis episodes, nasal congestion, reddening and swelling and in lowering allergen-specific immunoglobulin levels. These beneficial effects were obtained when the treatment was started prior to and extended during the allergen exposure season [75].

Despite these promising signs, early clinical trials on the impact of oral probiotic administration to asthmatic patients showed no significant differences in disease symptom severity or the number of asthma episodes. Early experiments demonstrated that yogurt supplemented with *L. acidophilus* had no impact on IgE and IL-4 levels or lung function in asthmatic patients. Other studies using two different *Lactobacillus* strains (*L. casei* and *L. rhamnosus*) confirmed these negative outcomes [76]. However, only a limited group of “good” bacteria has been tested to date, and different results might be obtained, for example, with bacteria known to produce SCFA or with a mix of bacteria and metabolites, which might determine a broader manipulation of the gut microbiota. It is suggested that this could be necessary to really induce the needed changes in an immature immune system, to counterbalance other predisposing factors and reduce the overall risk for pediatric asthma inception [58].

Another interesting approach to the use of whole bacteria is to rely on part of them to modify the airway bacterial communities. An example is given by the intranasal application of bacterial components (such as endotoxin or flagellin) that demonstrated immunomodulatory ability in the lung of different animal models reducing experimental asthma by inducing Tregs [15]. The concept of bacteriotherapy has been developed more than 70 years ago and recently applied in rRTIs in children. The strategy is based on the principle of bacterial interference, with pathogens colonization and growth inhibited by the commensals themselves and/or their production of antimicrobial proteins and peptides as well as immunomodulation. The strain of *Streptococcus salivarium* 24SMB has been used as a nasal spray in children with rRTIs, and it showed promising evidence. Confirmatory observations came from other bacterial species such as *Streptococcus oralis* 89a administered as an oral spray. Despite being interesting, the studies showed some methodological limitations and missed microbiological findings [77].

**Figure 8:**
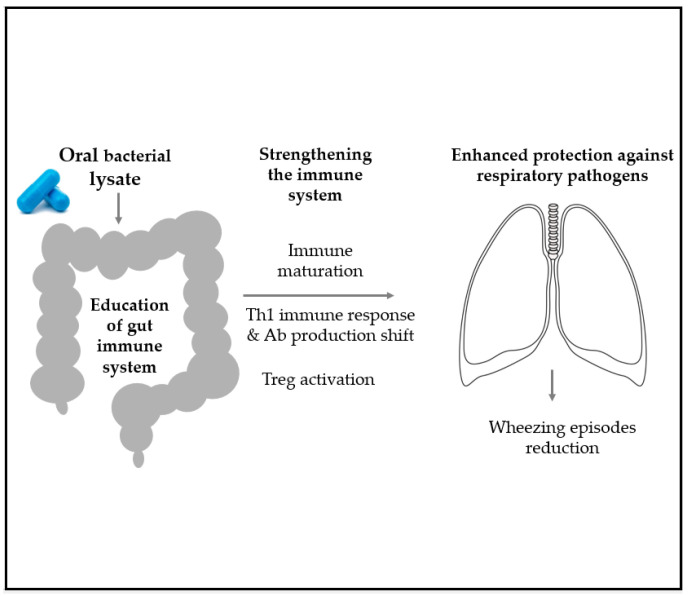
Oral lyophilized bacterial lysates (OBLs) could educate the gut immune system and contribute to the reduction of inflammation. In parallel, these compounds were shown to activate some pathways in the gut and provide the host with a broad protection against respiratory pathogens, via the migration of immune sensors and effectors from the GALT in the PPs to the MALT.

Oral lyophilized bacterial lysates (OBLs) derived by known heat-inactivated respiratory pathogens have gained interest in the medical community. Indeed, it has been hypothesized that they could mimic some gut microbiota profiles and functions, thus inducing specific immune molecular pathways that can lead to the activation of Treg and the reduction of type 2 inflammation. In parallel, these compounds have been shown to activate other pathways, leading to an increased broad protection against respiratory microbes. Their activity in the airways is based on the migration of immune sensors and effectors from the Gut Associated Lymphoid Tissue (GALT) of the PPs to the Mucosa Associated Lymphoid Tissue in the lung (MALT) via the lung-gut axis as shown in Figure 8 [23].

In an in vitro model, the bacterial extract OM-85 induced the production of the antimicrobial peptide β-defensin in bronchial epithelial cells This finding is of particular interest because recent evidence has underpinned a key role of host-defense peptides that include β-defensin in “farming” the microbiota and, consequently, in modulating the host cross-talks with commensal bacteria at different mucosal sites. In particular, altered expression of antimicrobial peptides has been described as contributing to disease state [78]. Some pre-clinical study findings and other mechanistic investigations conducted in children with bacterial lysates shed lights on other aspects of their mode of action in asthma. In detail, they have shown the ability of these products to decrease airway eosinophilia and bronchial hyper-responsiveness by the activation of Treg cells in the gut and by the induction of their migration to the airway mucosa in animal models of allergic inflammation [79,80]. Furthermore, at oral doses lower than those used in infants, they have shown to significantly decrease the concentration of both IL-5 and IL-13 in BAL of OVA-mice model [81,82]. In other experiments, they have shown to be able to increase the production of type I interferons (INF-α and INF-β), cytokines that are fundamental for an efficient anti-viral innate immune response [83]. The mechanism of action of OBLs was reviewed in a publication by Esposito et al. that also described the immunomodulatory features and the consequent clinical benefit in pediatric wheezing and asthma as well as in respiratory recurrences [18]. Some recent studies were aimed at better understanding the link between OBLs’ clinical benefit, their effect on the microbiota composition both in the gut and in the airways and their role in the complex inter-talk between microbiota and a developing immune system. These studies had the ultimate objective to show reduction of airways infection and associated wheezing and potentially to point out their putative role as disease modifiers if given as an early intervention [84,85]. OM-85 administration was reported to be associated with a significant change in the microbial composition in stools. Animals treated with OM-85 showed increased presence of bacteria from the genus *Lactobacillus* as compared with animals administered with phosphate buffered saline (PBS). Two *Lactobacillus* species, *L. reuteri* and *L. salivarius*, were identified as particularly abundant in animals treated with OM-85. An interesting hypothesis is that OM-85 can exert its effects by creating the conditions within the mucosal-microbiome interface for the growth of *Lactobacilli*, which in turn have been shown to modulate airway inflammation in animal models of asthma. Indeed, administration of *L. reuteri* to mice increased the activation of non-antigen-specific CD4+ CD25+ Foxp3+ regulatory T cells, which in turn attenuated allergic airway responses [86]. In summary, it has been hypothesized that OM-85 might induce a re-organization of gut and lung microbial communities, thus improving cross-talks with immune effectors that favor immune homeostasis and reduce disease exacerbations [87]. BLs could be used also intranasal as therapeutic approach against viral infections. This was shown in a model where mice pre-treated with aerosol of BLs exhibited faster and more effective inflammatory responses after IV, limiting tissue damage and improving the survival of the animals. This might be due to the immunomodulation that can control the cytokines storm after IV. Similar examples have come from the use of *Lactobacillus rhamnosus* used prior to RSV challenge that has been reported to limit the pulmonary destruction protecting against the deleterious effects of excessive inflammation [88].

It is still a long way to go before using bacterial-derived products for microbiome specific enrichment to prevent respiratory tract infections or their complications in humans. Confirmations in patients are needed and studies are still on-going [23]. Table 1 summarizes all the registered pediatric studies that in addition to clinical endpoints will investigate the effects of the OBL OM-85 on the host microbiota in children affected by rRTIs and wheezing episodes.

A further interesting consideration is that BLs might also play a role in trained-innate immunity, as described for some whole bacteria vaccines (i.e., BCG) [89]. Indeed, BLs might do this directly and/or by shaping the host microbiota, to provide a heterologous protection against a broad range of microbes. This leads to an enhanced and long-lasting boosted non-specific response to infections. This could be of particular interest not only in some pediatric populations, but also in patients with comorbidities and elderly that show a defective innate anti-viral response and that are at high risk for severe viral infections including SARS-CoV-2. In the present context of COVID-19 pandemic that affects both the respiratory and the intestinal tract in children, preserving the host microbiota or complement its functionality with probiotics or BLs might mitigate SARS-CoV-2 effects. No evidence is currently available, but this intuitive thinking is intriguing and has triggered the interest of the medical community [90] as well as that of researchers.

## 6. Conclusions

Respiratory infections and complications such as asthma are a burden in childhood and a challenge in clinical practice despite the identification of risk factors and the progress in prevention and care. Understanding of pathophysiology and developing of therapeutics. A lot still needs to be done to understand the pathophysiology of the different clinical entities and to develop prophylactic measures to prevent exacerbation recurrence and asthma inceptions in children. New sequencing techniques and advances in immunology have led to a better understanding of the links between the gut and airway microbiota disturbances and the impairment of the immune system against respiratory pathogens. The complex inter-talks between commensal microorganisms in the gut and immune and non-immune cells in the intestine have been unraveled as well as the fact that gut and airway microbiota might function as a “single organ.” Harmless microbes, their metabolites, such as SCFA, and/or their components have been used orally to prevent RTIs. Local use of whole bacteria strains or microbial-derived products have also been suggested. In particular, there is some preliminary evidence of oral and intranasal bacteriotherapy in children to prevent and reduce severity of both viral and bacterial infections. OBLs such as OM-85, one of the most studied, have a longstanding clinical evidence in prevention of rRTIs and, in pre-clinical models, have shown broad anti-viral and anti-bacterial features associated with anti-inflammatory and immunomodulatory properties. The immunomodulatory effect is aimed at correcting the Th1/Th2 balance in children to prevent respiratory infections and reducing associated complications, such as recurrent asthma episodes. Furthermore, they have been proposed as being able to re-arrange gut and airway microbiota. Because of the direct effects on the innate immunity and/or because of their “farming” effect on the host microbiota, these products might train a broad and long-term anti-viral innate immunity. This effect would be relevant in children and also in elderly and fragile adults in particular in the context of the COVID-19 pandemic.

## Figures and Tables

**Table 1:** Studies with OM-85 bacterial lysates investigating mechanistic endpoint including the effect on children microbiota.

Study Acronym	Indication	Patients/Children	Registry Number
OMPER	Prevention of RTIs	288	EudraCT2016-002705-19
ORBEX	Prevention of LWRI	>800 ^1^	NCT02148796
POWER	Prevention of severe LWRI	200	ACTRN12619000864123
CIRCAN	Prevention of recurrent LWRI requiring hospitalization	2268	ACTRN12620001370998

^1^ Original sample size 926.

## Data Availability

Data sharing not applicable.
